# Supplementary figures and images for: Responses of fungal communities at different soil depths to grazing intensity in a desert steppe
Source: PeerJ. 2025 Jan 6;13:e18791. doi: 10.7717/peerj.18791 (PMC11716020; doi:10.7717/peerj.18791)

# Spearman Correlation Plot

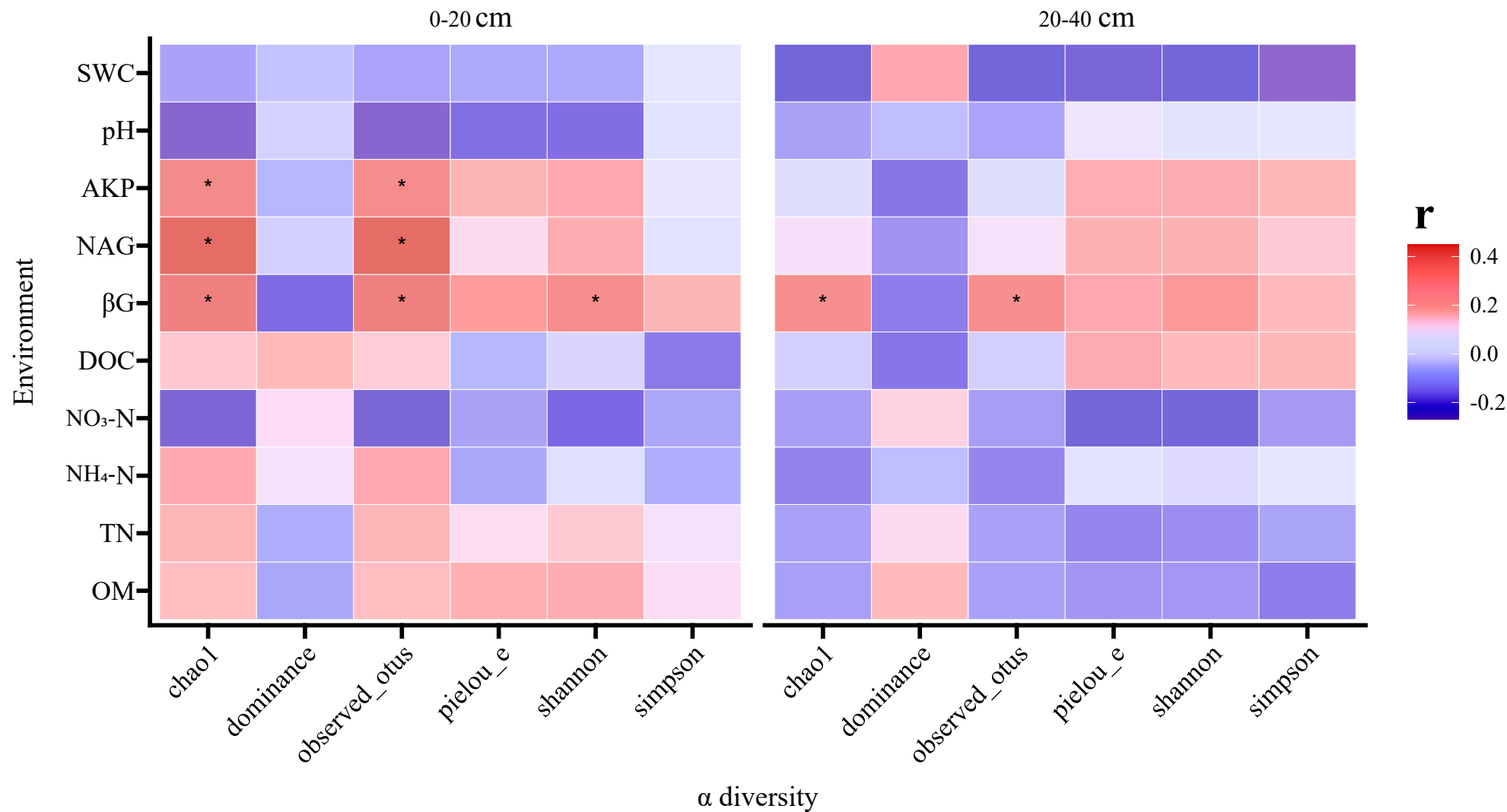

Supplement: Figure S1 — Spearman’s correlation analysis between soil physicochemical properties and fungal α diversity at two soil depths; red represents positive correlation and blue represents negative correlation. *Significant at the 0.05 probability level. **Significant at the 0.01 probability level. ***Significant at the 0.001 probability level. [file peerj-13-18791-s003.pdf]
