# Supplementary material for: Responses of fungal communities at different soil depths to grazing intensity in a desert steppe
Source: PeerJ. 2025 Jan 6;13:e18791. doi: 10.7717/peerj.18791 (PMC11716020; doi:10.7717/peerj.18791)
Supplement: Table S1 [file peerj-13-18791-s004.docx]

| **Table S1. The responses of fungal genera relative abundance to different grazing intensities at two soil depths.** | | | | | | | | | |
| --- | --- | --- | --- | --- | --- | --- | --- | --- | --- |
| **Method** | **Soil depth** | **Genus** | **NG** | **LG** | **MG** | **HG** | **OG** | ***H*** | ***P*** |
| K-W test | 0-20  cm | *Coprinopsis* | 0.0567b | 0.0042ab | 0.0013ab | 0.0003a | 0.0007a | 14.003 | 0.007 |
|  |  | *Clitocella* | 0.0542b | 0a | 0a | 0a | 0a | 25.773 | <0.001 |
|  |  | *Limonomyces* | 0.0107b | 0.0001a | 0.0098a | 0.0013ab | 0.0002ab | 16.407 | 0.003 |
|  |  | *Scutellinia* | 0.0096a | 0.0462a | 0.0572a | 0.0096a | 0.0310a | 12.082 | 0.017 |
|  |  | *Ceratobasidium* | 0.0093a | 0.0882b | 0.0326ab | 0.0554b | 0.0381ab | 17.742 | 0.001 |
|  |  | *Cercophora* | 0.0088a | 0.0061ab | 0.0023ab | 0.0026ab | 0.0014b | 11.016 | 0.026 |
|  |  | *Alternaria* | 0.0087a | 0.0049a | 0.0023a | 0.0124a | 0.0060a | 10.494 | 0.033 |
|  | 20-40cm | *Coprinopsis* | 0.0336b | 0.0038ab | 0.0012ab | 0.0016ab | 0.0009a | 13.353 | 0.01 |
|  |  | *Clitocella* | 0.0134b | 0a | 0a | 0a | 0a | 22.955 | <0.001 |
|  |  | *Darksidea* | 0.0221b | 0.0020a | 0.0078ab | 0.0104ab | 0.0087ab | 11.653 | 0.02 |
|  |  | *Limonomyces* | 0.0044b | 0a | 0.0001ab | 0.0003ab | 0.0002ab | 13.524 | 0.009 |
|  |  | *Scutellinia* | 0.0043ab | 0.0067ab | 0.0718b | 0.0025a | 0.0239ab | 14.052 | 0.007 |
|  |  | *Cercophora* | 0.0175b | 0.0094ab | 0.0046ab | 0.0017a | 0.0023ab | 14.559 | 0.006 |
|  |  | **Genus** | **NG** | **LG** | **MG** | **HG** | **OG** | ***F*** | ***P*** |
| Tukey | 20-40cm | *Mortierella* | 0.0321b | 0.0054a | 0.0182ab | 0.0120a | 0.0169ab | 4.547 | 0.007 |
|  |  | *Ceratobasidium* | 0.0209a | 0.0642ab | 0.0563ab | 0.1037b | 0.0465a | 5.207 | 0.003 |
|  |  | *Preussia* | 0.0080b | 0.0027a | 0.0027a | 0.0020a | 0.0037a | 5.874 | 0.002 |
|  |  | *Glomus* | 0.0060a | 0.0051a | 0.0073ab | 0.0117b | 0.0095ab | 5.017 | 0.004 |
| For each parameter, a different lowercase letter indicates a significant difference at the 0.05 probability level (*P* < 0.05) based on K-W tests (Levene’s test *p* < 0.05) or Tukey’s HSD (Levene’s test *p >* 0.05). Values in bold show statistically significant differences. The p-values of pairwise comparisons in the K-W test are corrected by the Bonferroni correction. No letter indicates no significant difference.  We used the top 20 relative abundance genera and did not show no significant difference.  NG: no grazing; LG: light grazing; MG: moderate grazing; HG: heavy grazing; OG: overgrazing. | | | | | | | | | |
