# Supplementary material for: Responses of fungal communities at different soil depths to grazing intensity in a desert steppe
Source: PeerJ. 2025 Jan 6;13:e18791. doi: 10.7717/peerj.18791 (PMC11716020; doi:10.7717/peerj.18791)
Supplement: Table S2 [file peerj-13-18791-s005.docx]

| **Table S2. Above-ground biomass (Agb), plant litter and coverage under different grazing intensities** | | | | | | | |
| --- | --- | --- | --- | --- | --- | --- | --- |
| **Treatment** | **NG** | **LG** | **MG** | **HG** | **OG** | **H** | ***P*** |
| Agb (g) | 131.65b | 37.57ab | 36.50ab | 35.40ab | 28.59a | 17.302 | 0.002 |
| Litter (g) | 29.37b | 2.22a | 3.89ab | 2.56a | 2.05a | 15.418 | 0.004 |
| Coverage(%) | 53.60c | 39.80bc | 31.20abc | 25.80ab | 23.40a | 21.815 | <0.001 |
| For each parameter, a different lowercase letter indicates a significant difference at the 0.05 probability level (*P* < 0.05) based on K-W tests. The p-values of pairwise comparisons in the K-W test are corrected by the Bonferroni correction.  NG: no grazing; LG: light grazing; MG: moderate grazing; HG: heavy grazing; OG: overgrazing. | | | | | | | |
