# Supplementary material for: Responses of fungal communities at different soil depths to grazing intensity in a desert steppe
Source: PeerJ. 2025 Jan 6;13:e18791. doi: 10.7717/peerj.18791 (PMC11716020; doi:10.7717/peerj.18791)
Supplement: Table S3 [file peerj-13-18791-s006.docx]

| **Table S3. Relative abundance of fungal phyla at two soil depths** | | | | |
| --- | --- | --- | --- | --- |
| Soil depth | 0-20 cm | 20-40 cm | T | Sig. (bilateral) |
| Ascomycota | 0.46173 | 0.50194 | -1.423 | 0.160 |
| Basidiomycota | 0.26736 | 0.24039 | 0.99 | 0.326 |
| Mortierellomycota | 0.01659 | 0.01834 | -0.454 | 0.651 |
| **Glomeromycota** | **0.04037** | **0.03215** | **2.351** | **0.022** |
| **Chytridiomycota** | **0.00256** | **0.00812** | **-2.096** | **0.044** |
| **Calcarisporiellomycota** | **0.00014** | **0.00062** | **-2.158** | **0.037** |
| Kickxellomycota | 0.00002 | 0.00004 | -1.253 | 0.216 |
| Rozellomycota | 0.00002 | 0.00002 | -0.08 | 0.937 |
| Olpidiomycota | 0.00001 | 0.00001 | 0.33 | 0.743 |
| For each parameter, a different lowercase letter indicates a significant difference at the 0.05 probability level (P < 0.05) based on T tests. Values in bold show statistically significant differences. | | | | |
