# Supplementary material for: Responses of fungal communities at different soil depths to grazing intensity in a desert steppe
Source: PeerJ. 2025 Jan 6;13:e18791. doi: 10.7717/peerj.18791 (PMC11716020; doi:10.7717/peerj.18791)
Supplement: Table S4 [file peerj-13-18791-s007.docx]

| **Table S4. Relative abundance of dominant fungal phylum at different grazing intensities** | | | | | | | | |
| --- | --- | --- | --- | --- | --- | --- | --- | --- |
| **Soil depth** | **phylum** | **NG** | **LG** | **MG** | **HG** | **OG** | ***F*** | ***P*** |
| 0-20 cm | Ascomycota | 0.530 | 0.405 | 0.545 | 0.474 | 0.555 | 1.832 | 0.154 |
|  | **Basidiomycota** | **0.292a** | **0.322 a** | **0.178a** | **0.207a** | **0.203a** | **2.813** | **0.047** |
|  | **Mortierellomycota** | **0.034 c** | **0.006a** | **0.022bc** | **0.013a** | **0.018ab** | **5.581** | **0.002** |
|  | **Glomeromycota** | **0.030ab** | **0.021a** | **0.028ab** | **0.045c** | **0.038bc** | **4.952** | **0.004** |
| 20-40 cm | Ascomycota | 0.483 | 0.455 | 0.492 | 0.427 | 0.451 | 0.384 | 0.818 |
|  | Basidiomycota | 0.324 | 0.283 | 0.213 | 0.271 | 0.245 | 0.860 | 0.501 |
|  | Mortierellomycota | 0.024 | 0.009 | 0.026 | 0.009 | 0.016 | 1.573 | 0.212 |
|  | Glomeromycota | 0.046 | 0.040 | 0.035 | 0.043 | 0.037 | 0.557 | 0.696 |
| For each parameter, a different letter indicates a significant difference at the 0.05 probability level (P < 0.05) based on Tukey’s HSD. F and P values in bold show statistically significant differences. Lowercase letters indicate differences between different grazing intensities. No letter indicates no significant difference. | | | | | | | | |
