# Supplementary material for: Responses of fungal communities at different soil depths to grazing intensity in a desert steppe
Source: PeerJ. 2025 Jan 6;13:e18791. doi: 10.7717/peerj.18791 (PMC11716020; doi:10.7717/peerj.18791)
Supplement: Table S5 [file peerj-13-18791-s008.docx]

| **Table S5. α diversity indices** | | | | | | |
| --- | --- | --- | --- | --- | --- | --- |
|  | |  | **Levene-test** | | **T-test** | |
|  | |  | **F** | **P** | **T** | **P** |
| Chao1 | | **NG vs LG** | **0.026** | **0.875** | **3.536** | **0.005** |
|  |  | **NG vs MG** | **0.229** | **0.642** | **3.419** | **0.007** |
|  |  | **NG vs HG** | **1.682** | **0.224** | **2.267** | **0.047** |
|  |  | NG vs OG | 2.448 | 0.149 | 2.01 | 0.072 |
| Observed_otus | | **NG vs LG** | **0.014** | **0.909** | **3.501** | **0.006** |
|  |  | **NG vs MG** | **0.311** | **0.589** | **3.46** | **0.006** |
|  |  | **NG vs HG** | **1.742** | **0.216** | **2.256** | **0.048** |
|  |  | NG vs OG | 2.399 | 0.152 | 2.01 | 0.072 |
| Shannon | | **NG vs LG** | **8.664** | **0.015** | **2.386** | **0.046** |
|  |  | NG vs MG | 1.773 | 0.107 | 0 | 0.998 |
|  |  | NG vs HG | 3.588 | 0.087 | 1.846 | 0.095 |
|  |  | NG vs OG | 0.057 | 0.816 | 2.478 | 0.033 |
| NG | 0-20 cm vs 20-40 cm | **cCao1** | **1.177** | **0.303** | **2.676** | **0.023** |
|  |  | **Observed_otus** | **1.203** | **0.298** | **2.649** | **0.024** |
| No t-test analysis of α diversity showed data without significance. Bold indicates p < 0.05 and the letter in Figure 1 are annotated following pairwise comparisons. | | | | | | |
