# Supplementary material for: Responses of fungal communities at different soil depths to grazing intensity in a desert steppe
Source: PeerJ. 2025 Jan 6;13:e18791. doi: 10.7717/peerj.18791 (PMC11716020; doi:10.7717/peerj.18791)
Supplement: Table S6 [file peerj-13-18791-s009.docx]

| **Table S6.A two-way ANOVA of α diversity indices** | | | |
| --- | --- | --- | --- |
|  |  | **F** | **P** |
| Grazing | **Chao1** | **2.937** | **0.029** |
|  | **Observed_otus** | **2.934** | **0.030** |
|  | Pielou_e | 1.131 | 0.353 |
|  | Shannon | 1.808 | 0.142 |
|  | Simpson | 0.865 | 0.491 |
| Depth | Chao1 | 3.452 | 0.069 |
|  | Observed_otus | 3.333 | 0.074 |
|  | Pielou_e | 0.113 | 0.738 |
|  | Shannon | 0.036 | 0.851 |
|  | Simpson | 1.800 | 0.186 |
| Grazing * Depth | Chao1 | 0.637 | 0.639 |
|  | Observed_otus | 0.627 | 0.646 |
|  | Pielou_e | 1.260 | 0.298 |
|  | Shannon | 1.234 | 0.309 |
|  | Simpson | 1.402 | 0.247 |
| Values in bold show statistically significant differences | | | |
